# Supplementary material for: Prevalence and Transmission of Extended-Spectrum Cephalosporin (ESC) Resistance Genes in Escherichia coli Isolated from Poultry Production Systems and Slaughterhouses in Denmark
Source: Antibiotics (Basel). 2023 Nov 8;12(11):1602. doi: 10.3390/antibiotics12111602 (PMC10668726; doi:10.3390/antibiotics12111602)
Supplement: Supplementary file 1 [file antibiotics-12-01602-s001.zip › Supplemental Material_ESC E. coli prevalence 111023.pdf]

Supplemental Material

Prevalence and transmission of extended-spectrum cephalosporin (ESC) resistance genes in *Escherichia coli* isolated from poultry production systems and slaughterhouses in Denmark

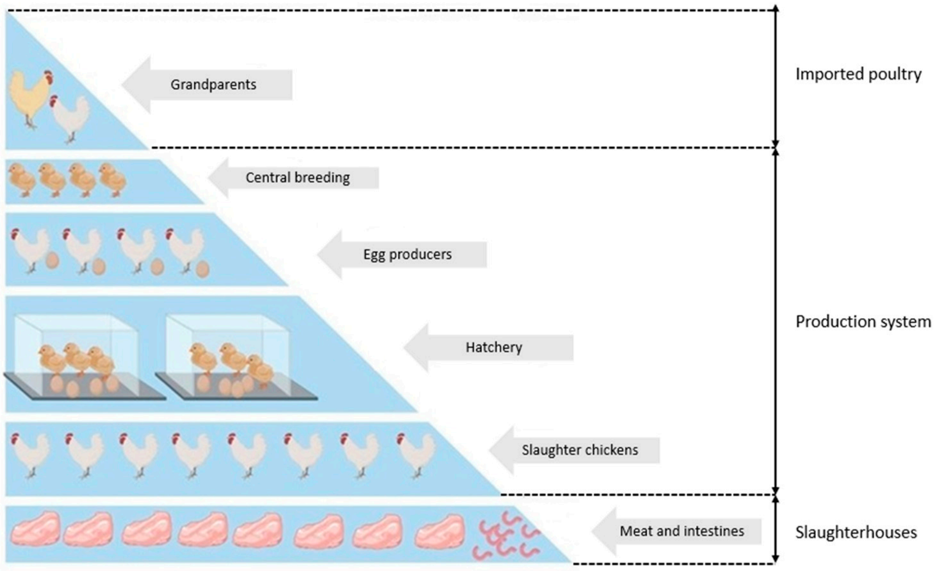

Figure S1. The conceptual structure of the Danish poultry pyramid. The imported chicks are off springs of grandparent birds. The chicks are reared to mature parent birds in the central breeding facility. At the egg-producing sites, mature parent birds are transferred for production of fertilized eggs, which are then moved to the hatcheries (hatching of chicks). Finally, the growth of the hatched chicks into full-sized slaughter chickens take place on the farms.

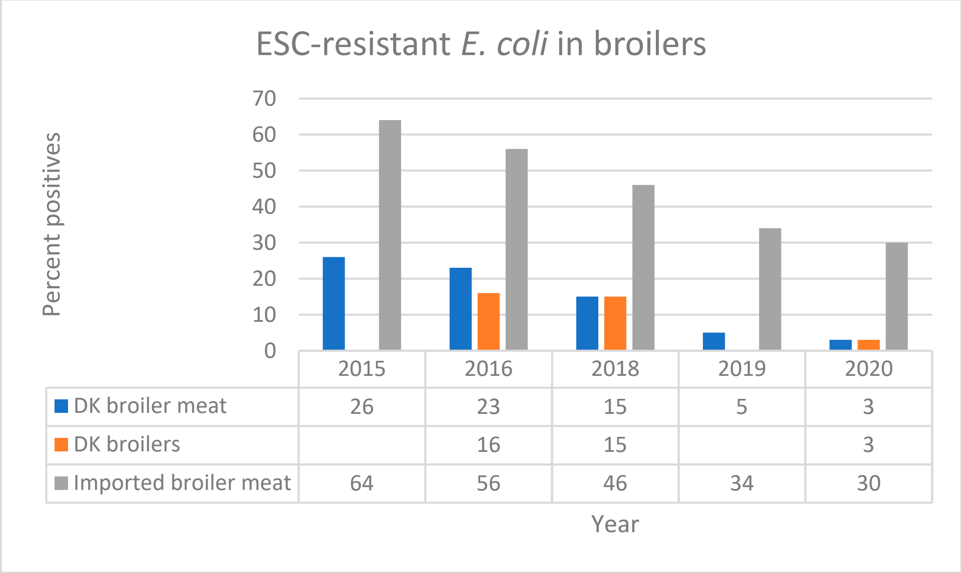

Figure S2. Prevalence of ESC-resistant *Escherichia coli* in domestic and imported poultry products on the Danish market from 2015 to 2020 according to the Danish surveillance program (DANMAP). Within the DANMAP surveillance program, samples of broiler meat and broilers are randomly collected at retail stores and slaughterhouses, respectively, by inspectors from the Danish Veterinary and Food Administration. The data can be accessed at https://www.danmap.org/
